# The Potential Role of COVID-19 in the Pathogenesis of Multiple Sclerosis—A Preliminary Report

**DOI:** 10.3390/v13102091

**Published:** 2021-10-17

**Authors:** Noothan J. Satheesh, Salam Salloum-Asfar, Sara A. Abdulla

**Affiliations:** Neurological Disorders Research Center, Qatar Biomedical Research Institute (QBRI), Hamad Bin Khalifa University (HBKU), Qatar Foundation (QF), Doha 34110, Qatar; noothanjyothi@yahoo.co.in

**Keywords:** coronavirus, COVID-19, SARS-CoV-2, neuroinflammation, multiple sclerosis

## Abstract

Coronavirus 2019 (COVID-19) is an infectious respiratory disease caused by severe acute respiratory syndrome coronavirus 2 (SARS-CoV-2) that mainly affects the lungs. COVID-19 symptoms include the presence of fevers, dry coughs, fatigue, sore throat, headaches, diarrhea, and a loss of taste or smell. However, it is understood that SARS-CoV-2 is neurotoxic and neuro-invasive and could enter the central nervous system (CNS) via the hematogenous route or via the peripheral nerve route and causes encephalitis, encephalopathy, and acute disseminated encephalomyelitis (ADEM) in COVID-19 patients. This review discusses the possibility of SARS-CoV-2-mediated Multiple Sclerosis (MS) development in the future, comparable to the surge in Parkinson’s disease cases following the Spanish Flu in 1918. Moreover, the SARS-CoV-2 infection is associated with a cytokine storm. This review highlights the impact of these modulated cytokines on glial cell interactions within the CNS and their role in potentially prompting MS development as a secondary disease by SARS-CoV-2. SARS-CoV-2 is neurotropic and could interfere with various functions of neurons leading to MS development. The influence of neuroinflammation, microglia phagocytotic capabilities, as well as hypoxia-mediated mitochondrial dysfunction and neurodegeneration, are mechanisms that may ultimately trigger MS development.

## 1. Introduction

**Figure 1:**
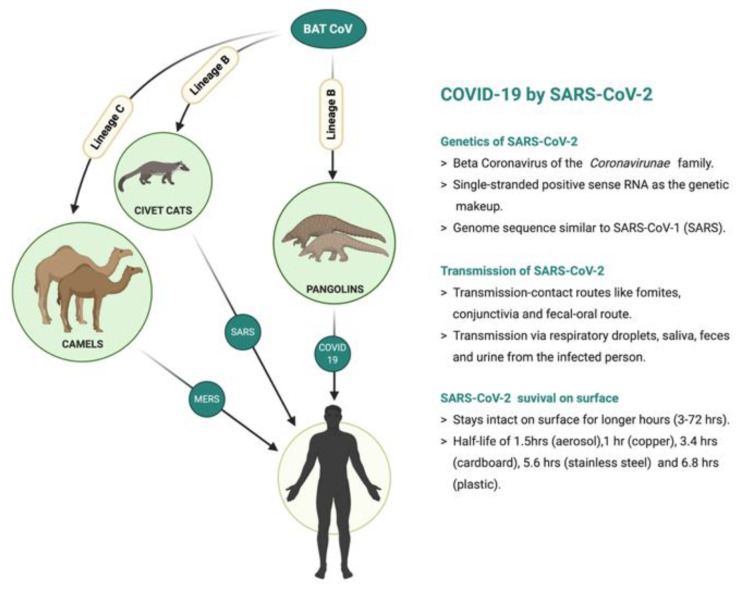
Different types of coronavirus infections: their sources and intermediate hosts—SARS-CoV-1 (SARS), MERS CoV (MERS), and SARS-CoV-2 (COVID-19). Figure details on SARS-CoV-2; its genetics, transmission, and survival on various surfaces. Created with BioRender.com. Agreement number: SW232PTQT3.

The coronavirus disease 2019 (COVID-19), caused by severe acute respiratory syndrome coronavirus 2 (SARS-CoV-2), has affected more than 174.9 million globally [1]. During the past two decades, three significant coronavirus outbreaks have been identified—SARS (November 2002), MERS (June 2012), and COVID-19 [2]. COVID-19 is the more infectious strain which prompted countries to enter lockdown with hampered trade, tourism, and education along with a quick expansion in health care systems to adjust to the increased scale of infected individuals and fatalities with an average death rate of around 2.16%. About 2.1 billion vaccine doses have been administered up to 10 June 2021 [1] (Figure 1).

COVID-19 exhibits highly heterogenous respiratory symptoms ranging from hypoxia cases associated with respiratory failure—acute respiratory distress syndrome (ARDS)—to minor symptoms or asymptomatic conditions [3]. Significant clinical symptoms caused by SARS-CoV-2 in COVID-19 include pneumonia, lower respiratory symptoms such as a cough and shortness of breath [4], fever, fatigue, and in some cases, it causes less common symptoms such as headaches, sputum production, diarrhea, and upper respiratory tract symptoms such as coryza breath [5,6,7]. Apart from the direct effect of SARS-CoV-2 on the lungs, these viruses tend to impact the central nervous system (CNS) [7,8]. Evidence has shown that post-COVID syndrome includes brain fog and chronic fatigue syndrome [9,10] and about 33.62% of 236,379 COVID-19 patients showed neurological or psychiatric issues for the first time, which is alarming [11]. Thus, it is important to understand the neuro-invasiveness and neurotropic nature of SARS-CoV-2. Moreover, it is essential to note that cases that exhibit headaches, a loss of smell and taste, confusion, dizziness, and impaired consciousness highlight an essential and influential link between SARS-CoV-2 infection and the CNS [8,9,12,13]. A recent study on COVID-19 differentially expressed genes confers an association with Multiple Sclerosis (MS) development in the future [10]. Interestingly, previous studies have also shown an association of coronavirus with MS [13]. The Mouse Hepatitis Virus (MHV), a murine coronavirus-induced model, is a widely used in vivo model used to understand the demyelination mechanisms associated with MS. This review emphasizes the possible neuro-invasive route of SARS-CoV-2 and its association with encephalitis, encephalopathy, acute disseminated encephalomyelitis (ADEM), and the possibility of developing MS and other neurological diseases as a secondary effect due to SARS-CoV-2 infection.

## 2. Mechanisms of SARS-CoV-2 Invasion and the Effects on the Nervous System

The mode of zoonotic transfer of coronavirus from bats to humans in SARS, MERS, and COVID-19 is via an intermediate host such as civet cats, camels, and pangolins, respectively (Figure 1) [14]. SARS and SARS-CoV-2 enter humans via ACE2 receptors, mainly expressed in the lungs, brain, heart, blood vessels, gut, kidney, and testis [15]. Computational analysis has suggested that the zoonotic transfer of the SARS-CoV-2 virus occurs via a binding mechanism between ACE2 [16] and TMPRSS2 [17,18]. Apart from the generic respiratory complications caused by a SARS-CoV-2 infection, a plethora of evidence has supported the potential effect of SARS-CoV-2 on both the CNS and the peripheral nervous system (PNS) [13,14,19,20]. The effects of SARS-CoV-2 infections on the CNS include headache, loss of consciousness, vertigo, acute cerebrovascular disease, loss of muscle control (ataxia), and seizures, while the effects on the PNS include loss of smell, taste, vision, and episodes of neuropathic pain [19]. A recent study pointed out that the symptoms of SARS-CoV-2 go far beyond the respiratory and sensorial dimensions and involve psychosensorial and neurological dimensions. Many of these neurological symptoms were present in 78 out of 214 hospitalized COVID-19 cases (36.4%) [21]. Furthermore, another study that included 1099 patients with SARS-CoV-2 infection showed that they also suffered muscle pain, encephalitis, encephalopathy, epileptic seizures, stroke, rhabdomyolysis, and Guillain-Barre syndrome [22,23]. Moreover, genome sequencing confirmed the presence of SARS-CoV-2 in the cerebrospinal fluid (CSF) of infected persons, proving the entry of SARS-CoV-2 and the effect on the CNS [24]. The following subsections will explain the potential neurological complications implicated in SARS-CoV-2 infection.

Neurological complications of SARS-CoV-2 infection are associated with encephalitis, encephalopathy, and ADEM. Several studies have concluded that SARS-CoV-2 is associated with encephalitis and encephalopathy, with a potential effect of viral infection on the CNS of these patients [25]. Encephalitis, the inflammation of the brain, is caused by direct infection by viruses known as acute encephalitis or due to an immune response corresponding to an infection known as ADEM. Acute encephalitis appears within days or periods of one or two weeks, interferes with the patient’s consciousness, and shows symptoms of headache, lack of orientation, and neurological issues [26]. ADEM is a rare demyelinating disease of the CNS which progresses rapidly with autoimmune processes followed by infection via viral exposures or immunization [27,28,29]. ADEM is associated with fever, meningitis, seizures, and unconsciousness. It is generally seen in children rather than adults [27,28,29], with a slight predominance in females rather than males [28]. Another well-known neurological complication is encephalopathy, which is a reversible brain dysfunction caused by metabolic disorders, systemic toxemia, or hypoxia during the acute infection generally characterized by cerebral edema. Patients with infectious toxic encephalopathy display headaches, mental disorders, disorientation, paralysis, loss of consciousness, and coma. Interestingly, COVID-19 patients have also reported viremia, severe hypoxia, and lately associated encephalopathy [24,30,31]; however, more studies are still needed to understand the pathophysiology behind the development of viral encephalitis or encephalopathy associated with SARS-CoV-2 infection in COVID-19.

**Figure 2:**
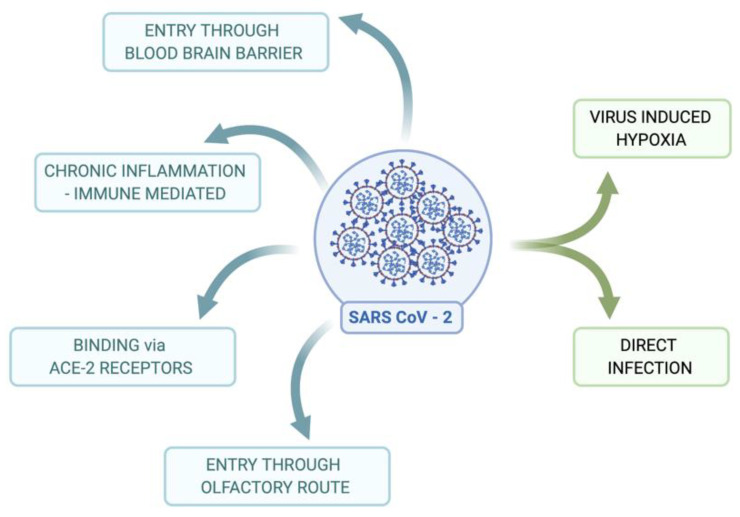
Possible routes of entry of SARS-CoV-2 to the brain to cause infection: Blue color: confirmed entry routes. SARS-CoV-2 binds to ACE-2 receptors in humans, migrates via the olfactory route, and crosses the BBB to enter the CNS to cause brain infection. SARS-CoV-2 could also mediate via an immune-mediated pathway to enter the CNS. Green color: Route that needs further study in association with SARS-CoV-2 includes virus-induced hypoxia and direct infection to the brain. Created with BioRender.com; Agreement number: LO232PSOFU.

Neuro-invasive and neurotropic SARS-CoV-2 exerts different infection stages, starting with a marked loss of smell and or taste during the early stages of infection to immunomodulatory effects affiliated with seizures at later stages [25]. Even though the brain is highly guarded and protected by the blood–brain barrier (BBB), and blood–CSF barriers protect the brain from the entry of external molecules, pathogens, and cells, the permeability of the BBB is strictly controlled by tight junctions (TJ) by continuous capillaries with no fenestrations [32,33,34]. As neurotropic viruses such as SARS-CoV-2 enter the CNS from the primary infection site, they invade the nervous tissues and disrupt its homeostasis, causing infections [24]. Inflammation of the CNS, known as neuroinflammation, includes both immunological and neuronal cells and results in a modulation of the immune response of the nervous system and synaptic plasticity [35]. The entry of SARS-CoV-2 into the CNS could occur via the hematogenous or peripheral nerve routes. The hematogenous route would be the main route for the neuro-invasiveness of SARS-CoV-2 and could be facilitated by the Trojan Horse mechanism, a mechanism by which the pathogen infects the CNS by crossing the BBB through transcellular, paracellular, and/or via infected phagocytic cells [36], which most of the neurotropic viruses adapt to enter the CNS. Neuro-invasion is mediated by the olfactory neurons and is initiated at the olfactory epithelium via bipolar cells, with its axons and dendrites reaching the olfactory bulb resulting in the formation of synapses across the cells [32,37]. Studies in transgenic rodent models that express human ACE2 have confirmed the transfer of coronaviruses from the nasal cavity to the CNS. The SARS-CoV viral antigen was detected in the olfactory bulb after 60 h and the brainstem after four days upon the intranasal administration of SARS-CoV in K18-hACE2 transgenic mice [37,38]. A recent study by Dube et al. in 2018 also confirmed coronavirus’s entry to the CNS via the olfactory bulb [37,39]. The virus’s entry to the CNS alters the neurons and marks the initial step for disease progression with its neurotropic nature and the associated immune response [37,40]. Collectively, this depicts the potential and impactful neurotropic influence of SARS-CoV-2 within the nervous system [37,41] (Figure 2).

## 3. MS and Coronavirus Infection

Shreds of evidence suggest that the coronavirus infection could be associated with MS. Clinicians differentiate ADEM from MS as an unrepeated monophasic incident while the latter is considered as a relapse or as progressive disease [42]. MS is a classic example of a demyelinating disease characterized as an autoimmune inflammatory disease with chronic demyelination of the white matter with unidentified etiology [43,44]. Some of the clinical manifestations associated with MS include fatigue, muscle spasms, depression, cognitive dysfunction, seizures, focal sensory loss, vertigo, ataxia, and trigeminal neuralgia. Demyelination is generally referred to as the ways in which the myelin sheaths around the axons are lost/removed, occurring in both the CNS and PNS. Demyelination affects memory function, and the survival of neurons as demyelination in the hippocampus leads to a reduced expression of the neuronal genes. This, in turn, affects axonal transport with decreased synaptic density, glutamate receptors, and reduced intermediates [44]. Besides, demyelination also results in neurodegeneration development [43,45]. In MS, due to demyelination, continuing irreversible decline in neurological function occurs with progressive axonal damage due to the loss of the connection between the axons and the myelin sheath [43,46]. This leads to axons with swelling, a reduced caliber, and degeneration with continual advancement in the development of MS [43]. Demyelination is reversed by spontaneous remyelination by the oligodendrocytes, and its balance is mainly maintained by both innate and adaptive immune systems determining the effect and severity of the demyelinating disease. Neuronal damage in MS is mostly associated with excitotoxicity via glutamate, which significantly increases in the CSF and brain of MS patients [47]. In MS, the inflammatory systems are linked directly to synaptic dysfunction in the hippocampus, which includes eliminating the synapses with the complement system’s activation [48]. 

The initial MS presentation occurs between 15 and 55 years and is mainly reported in women rather than in men [49]. In its early development stage, inflammatory cell-induced demyelination is associated with different processes such as the activation of the microglia, oxidative stress, and damage of the mitochondria in the axons. This process amplification depends on the brain complexity due to aging (i.e., iron accumulation in the brain with aging). The damage of the mitochondria in the axons leads to prolonged stress in the cells and the loss of ionic homeostasis, leading to the death of axons and neurons. A closer look into MS and animal models of MS has shown that with aging and disease progression, the cell remyelinating capacity deteriorates, leading to a worsening of the disease [50].

Mouse models and studies associated with epidemiological analysis and identical twins confirmed the association of MS with viral infections [51]. Earlier studies in 1980 have shown coronavirus-like particles in patients with MS [52]. For this, histologic analysis performed on the brain tissues collected from autopsies of MS patients showed a demyelinated area surrounded with astrocytes. These brain tissues were inserted into the intracerebral area of weaning mice. Later, the coronavirus-like particle was confirmed in the cell-culture systems by using the livers and brain of the infected suckling mice [52]. This finding was later supported by a study in 1992, in which the viral RNA of the human coronavirus (HCV) 229E was detected in the CNS tissues of four MS patients [53]. The presence of ribonucleic acids (RNAs) in the CSF and the antibodies of human coronavirus have also been detected in MS patients, confirming an infection of the CNS following a coronavirus infection [54]. Apart from the immediate effect of a viral infection, the virus could stay in the body in a dormant phase, and a latent phase of the virus would be followed by a reactivation of its viral activity and would lead to oligodendrocytes lysis to progressive multifocal leukoencephalopathy or demyelination, a condition usually associated with coronavirus infections [51]. Hence, as a long-term effect of SARS-CoV-2 infection on CNS, the possibility of MS cannot be ruled out.

Furthermore, MHV, a type of coronavirus that infects mice, has been widely used to understand the neurological manifestations of CNS and the development of MS upon coronavirus infection [54]. Administration of MHV to mice via an intracranial route induced acute severe encephalomyelitis in mice affecting the astrocytes, microglia, and oligodendrocytes. Although there were no viral loads detected in the animals that survived after two weeks of administration, the oligodendrocytes expressed viral antigens in the survived mice. This shows the exertion of viral activity by MHV, with a progression of a demyelination disease mediated by several immune cells. MHV is considered as the perfect model to study MS pathogenesis, as it showed both demyelination and remyelination in mice models upon MHV infection, which is a vital characteristic in MS [44,55]. 

Both the intranasal and intravenous administration of MHV on mice and primates caused an infection of the CNS and confirmed the coronavirus’s neurotropic effect [54]. It is often associated with the downregulation of IFN-β in BMECs), causing acute encephalomyelitis and demyelination. Demyelination due to MHV infection involves the activation of microglia and immune cell-mediated inflammatory responses. Matias-Guiu et. al. provided in their study a potential base to understand the MS pathology by coronavirus infection [54]. Consequently, it is likely that the patients with SARS-CoV-2 infection would develop earlier, and delayed responses of neurological complications, with MS as a probable delayed manifestation [54].

In a recent study of COVID-19 confirmed cases, neurological manifestations were shown by the presence of oligoclonal bands with the same pattern in serum and elevated levels of proteins and immunoglobulins in CSF, a reliable indicator of MS [47,56]. Moreover, optic neuritis followed by SARS-CoV-2 infection with demyelinating lesions in the CNS has been reported [57]. This evidence could be an indication of MS development in the future. However, whether these bands were present before the SARS-CoV-2 infection needs to be further confirmed. Several studies have confirmed the potential role/presence of coronavirus in MS, and therefore, the potential effect of SARS-CoV-2 infection in MS development is possible. Its possible chances and mechanisms need to be further investigated.

Moreover, several reports of COVID-19 infected patients with a possible association with ADEM have been published, as cases of an immune-mediated effect on CNS that occurred after SARS-CoV-2 infection [27,58]. These studies confer a strong association of SARS-CoV-2 infection with ADEM and could be considered an early symptom in similar patients related to the development of MS in the future via a direct or indirect effect of the virus. ADEM is mostly monophasic, with rare relapsing cases and it is challenging to distinguish this from MS [28]. Clinically, MS and ADEM represent demyelinating autoimmune diseases that affect the CNS [59]. In a recent case study, it was observed that over three years, 35% of adult patients initially diagnosed with ADEM developed MS [28]. Furthermore, several studies have confirmed that the development of pediatric MS in children happened within years after ADEM occurrence [60,61]. Therefore, with the current understanding of SARS-CoV-2 infection, in addition to the development of encephalitis/encephalopathy and given that ADEM is an immediate neurological complication, one can hypothesize that MS could happen as a secondary effect of SARS-CoV-2 infection.

## 4. Possible Mechanisms for Viral/SARS-CoV-2 Infection-Mediated MS Development

The exposure of a genetically susceptible person to a potential viral trigger from the environment leads to a cascade of autoimmune responses leading to demyelination and MS development. These insults interrupt the balance between myelin antigens in axons (as myelin sheets that surround axons) or oligodendrocytes (myelin-forming cells) and T-cells [62]. Viral entry to the CNS could initiate the first stage of MS progression involving various types of cells, mainly innate and adaptive immune cells, and glial cells [63]. The innate immune cells respond to an external stimulus by recognizing the pathogen-associated molecular patterns (PAMPs) by PRRs, mainly by TLRs that are receptors expressed on the innate immune cells [63,64]. Effector mechanisms by the activated innate immune system include the production of nitric oxide and oxidative burst, the phagocytosis of nearby pathogens, apoptotic cells, and myelin sheaths, the production of chemokines and cytokines, antigen presentation to the adaptive immune cells, tropic factors secretion and the release of MMPs that disturbs the extracellular matrix and the BBB [63]. Signals from the innate immune system activate the adaptive immune system to expand the T-cells and B-cells [64]. 

With the exponential knowledge of SARS-CoV-2 infection and the presence/ability of coronavirus to develop MS in patients and in in vivo models, one may predict a future wave of MS similar to Parkinson’s disease following the influenza pandemic of 1918 (The Spanish Flu) [65]. Apart from coronavirus, herpes viruses such as the varicella-zoster virus (VZV), the herpes simplex virus (HSV-1 and HSV-2), the cytomegalovirus (CMV), the human herpes virus 6 (HHV-6), and the Epstein–Barr virus (EBV) are known as triggers in MS development (Table 1). Moreover, viruses such as the human polyomavirus 2 or John Cunningham virus (JCV) and the Human endogenous retroviruses (HERVs-H and W) are also associated with MS predisposition through the increase in neuroinflammation [66,67]. The following section discusses the possible mechanisms that could mediate MS development due to SARS-CoV-2 infection.

### 4.1. Cytokine Storm and Neuroinflammation

**Figure 3:**
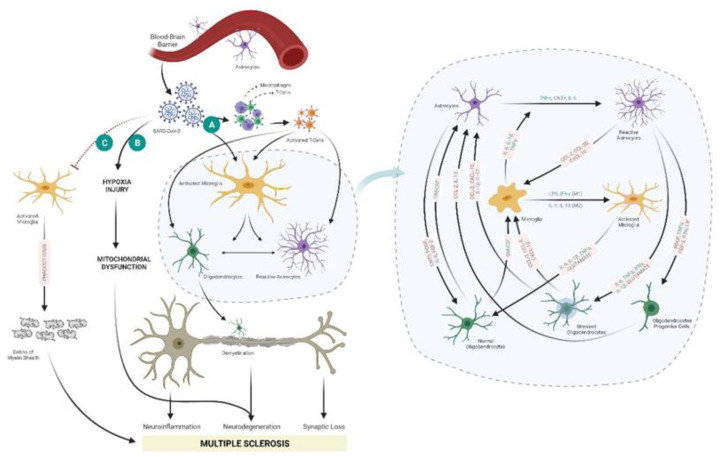
Possible ways by which SARS-CoV-2 leads to MS. Within the CNS, neurotropic and neurotoxic SARS CoV-2 would interfere with demyelination/remyelination, neurodegeneration, Neuroinflammation and synaptic loss of neurons leading to MS progression. Possible ways: (**A**) Cytokine storm and increased demyelination- entry of SARS-CoV-2 could activate immune cells (macrophages and T-cells) and glial cells, with increased expression of several cytokines, interleukins and chemokines thereby leading to demyelination [75,79,80,81]. (**B**) Hypoxia-induced mitochondrial dysfunction and (**C**) Reduced phagocytosis of myelin sheath debris, SARS-CoV-2 might decrease the phagocytic capacity of microglia cells, and macrophages of myelin sheath debris; accumulation of myelin sheath debris hinder the access of the remyelinating cells such as Schwann cells causing MS; Blue coded cytokines, interleukins, and chemokines represent the molecules involved in SARS-CoV-2 infection [70]. Created with BioRender.com; Agreement number: ZK232PROSX.

Clinical data confirm an association of an immunological effect of SARS-CoV-2 infection leading to a cytokine storm. A cytokine storm is characterized as a critical immune response which prompts the hyperactivation and proliferation of immune cells such as natural killer cells, macrophages, and T-cells [68] with glial cell activation in the CNS, causing neuroinflammation demyelination [25,27,69]. Cytokine profiling of COVID-19 samples mainly with patients admitted in the intensive care unit has shown an increase in IL-2, IL-7, GM-CSF, IFN- γ inducible protein 10 (IP-10; CXCL10), MCP-1, MIP-1, and TNF- α [69] which could cause viral-induced hyper-inflammation [69,70]. Moreover, severe cases of COVID-19 cases have shown an increase in IL-1β, IL-1ra, IL-2R, IL-6, IL-8 (CXCL8), IL-17, IFN-γ, and GM-CSF [70] (Figure 3/Table 2). 

In viral infections, this cytokine storm leads to the apoptosis of the lungs’ epithelial and endothelial cells, resulting in vascular leakage, hypoxia, and alveolar edema [68]. Upon a pathogenic insult to the organism, viruses such as SARS-CoV-2 itself or the cytokines could cross the BBB via transporters and circumventricular organs and activate the glial cells, mostly microglia initiating an intricate neuroinflammatory signaling cascade with the release of several cytokine and chemokines [35]. In the CNS, the infiltration of various immune cells and cytokines released from these cells leads to an inflammation of white and gray matter (Neuroinflammation), leading to MS development. This includes the association between the myelin-specific T helper (Th) cells and MHC class II, presenting alleles and antigen-presenting cells (APCs) [62,71]. The ligand-binding receptor of PAMPs in viruses or bacteria binds to TLRs expressed on the cell surface, leading to the release of various cytokines such as IL-4, IL-12, and IL-23. In the presence of these cytokines, CD4^+^ T cells differentiate into helper T-cells: Th1 (pro-inflammatory), Th2 (anti-inflammatory), or Th17 (pro-inflammatory) phenotypes releasing specific cytokines. Pro-inflammatory cytokines such as TNF-α and IFN-γ are released by the Th1 cells which suppress the differentiation of Th2 cells. The anti-inflammatory role of Th2 cells is exerted by IL-4 and IL-13, of which IL-4 decreases inflammation via activation and an increase in M1 and M2 (repair) macrophages. The anti-inflammatory role of IL-13 is exerted via the release of MMPs. IL-17, IL-21, IL-22, and IL-26 mediate the inflammatory response of the Th17 cells in MS [71]. CD4^+^ regulatory T cells (Treg) cells play a role in suppressing the excessive inflammatory responses by inhibiting the proliferative, functional and migrative capacity of the effector T-cells via secreting cytokines or cell-surface molecules. Treg cells also promote remyelination; however, the capacity of the Treg cells has been found to be altered in MS patients [72]. 

Neuroinflammation is associated with the release of numerous pro-inflammatory factors such as TNFα, IL-1β and nitric oxide free radicals leading to the subsequent recruitment of more macrophages and microglia to the CNS to remove the cell debris produced during the neural injury. This continual exposure of neurons to pro-inflammatory cytokines results in neuronal dysfunction and degeneration that is mainly associated with the development of age-related neurodegenerative diseases [73]. Activation of astrocytes by pro-inflammatory cytokines, stress (oxidative or chemical), pathogen-associated molecular patterns (PAMPs) leads to the expression or upregulation of cytokines (TNF-α, IL-6 and IL-1β), chemokines (CCL2, CCL20, and CXCL10), neurotrophic factors including nerve growth factor (NGF), brain-derived neurotrophic factor (BDNF), vascular endothelial growth factor (VEGF), and leukemia inhibitory factor (LIF), major histocompatibility complex (MHC)- class II cell adhesion molecules such as ICAM-1, VCAM-1 and TLRs [74,75]. These molecules play a crucial role in killing the invading pathogens; however, they also exert bystander damage to the adjacent glial cells and neurons [73] (Figure 3A).

Lesions of MS are associated with several demyelinated plaques within the white matter accompanied by a cluster of several inflammatory cells such as activated microglia, lymphocytes, and macrophages [43,44,76]. Inflammatory and neurotoxic responses in MS lesions by reactive astrocytes cause tissue damage via the manipulation of glutamate (increased) and redox homeostasis [74]. However, astrocytes play a central role in dampening the inflammation, thereby promoting neuroprotection and repairing lesions in MS [74]. Scattered plagues in MS formed due to demyelination are enclosed with reactive astrocytes and might exert emperipolesis, where the astrocyte engulfs one or more cells such as oligodendrocytes [77] or lymphocytes [78]. However, the role of emperipolesis in MS is yet not exact. Demyelination is also associated with cytotoxic T cells (CD8^+^ T cells) [62,71], which releases perforin-pore forming cytolytic protein that has defined roles in suppressing and inactivating T-helper cells (CD4^+^ T cells). Perforin promotes astrocyte activation, disrupts tight junction organization, and increases vascular permeability of CNS [62,71,79]. Perforin induces apoptosis in oligodendrocytes leading to repair of myelin sheath in the CNS [71]. Calcium ions could mediate this. In MS, oligodendrocytes are reduced in numbers and show signs of stress and apoptosis, swelling with complement deposition, and cell lysis. 

### 4.2. Hypoxia Mediated Mitochondrial Dysfunction and Neurodegeneration

Recent findings on the mitochondrial involvement in MS pathogenesis [82,83] are exciting, and these correlate with another probable secondary effects of SARS-CoV-2 infection. SARS-CoV-2 infection-associated encephalopathy is related to hypoxia [84], and hypoxia-induced mitochondrial dysfunction could be a possible mechanism for the progression of MS in these patients. Mitochondria play a vital role in regulating calcium and ATP synthesis and constitute a significant source of reactive oxygen species (ROS). Mitochondria have a key role in maintaining a cellular environment’s bioenergetics via KREBS’s Cycle and Oxidative phosphorylation, cell-signaling, calcium storage, and apoptosis [85]. In the CNS, the mitochondrial metabolic activity would also be associated with an impaired Krebs cycle or neuronal oxidative phosphorylation [82]. Mitochondrial dysfunction leads to intracellular dysregulation and lower energy production resulting in neuronal damage, which is highly dependent on ATP for the transmission of electric signals and interrupts the anterograde and retrograde transportation across the axons [86]. Therefore, as mitochondrial dysfunction is involved in MS development [85,86], there is a possibility that SARS-CoV-2 infection could lead to mitochondrial dysfunction and further accelerate progression to MS development (Figure 3B). However, this needs to be further investigated. 

### 4.3. Altering the Phagocytotic Capability of Microglia/Macrophage

SARS-CoV-2 could alter the demyelination/remyelination equilibrium by microglia and macrophages in the brain, and this could result in the accumulation of myelin sheath debris and MS development. Both microglia and macrophages are of myeloid origin and play a crucial role in phagocytosis. As the resident macrophage cells in the CNS, microglia mostly have a role in removing cell debris after ischemia or damage to the myelin sheaths [62,87] and this is a crucial process for efficient remyelination followed by the demyelination of axons. Microglial phagocytosis occurs during neuronal connection restructuring, acute CNS injury, MS, and ageing via three main mechanisms [88]:

(a) Phagocytosis of myelin and extracellular aggregates such as amyloid-β particles;
(b) Release of growth factor, neurotrophic factors, and anti-inflammatory cytokines would stimulate axon branching and repair myelin sheaths;
(c) Recruitment of stem cells and other precursor cells and the triggering of astrocytes to release trophic factors that would neurons to develop and maintain synaptic connections.

Accumulation of myelin sheath debris leads to the formation of a dense matrix surrounding demyelinated axons, thereby blocking the remyelinating cells to demyelination sites. Hence, the efficient removal of myelin sheath debris would ease the access of remyelinating cells to the demyelinated axons. This accumulation of myelin sheath debris could also affect remyelination by blocking the maturation of the oligodendrocyte progenitor cells. Studies in the demyelination model by Kotter et al. observed impaired remyelination with decreased macrophages and microglia with decreased removal of myelin sheaths debris [89,90]. Hence, a possible MS development mechanism due to SARS-CoV-2 infection could be via accumulation of myelin sheath debris due to fewer microglia/macrophages in CNS (Figure 3C). Thus SARS-CoV-2 could increase the stride of demyelination of axons in CNS leading to MS progression by interrupting the phagocytotic role of macrophages/ microglia and thereby hindering remyelination.

## 5. Conclusions

Currently, the world is in a race against the COVID-19 pandemic, with a significant focus on patient care and substantial research in developing and implementing strategies to eliminate the virus’s spread. Coronavirus is considered to be one of the most invasive viruses in history that can even invade brain cells directly. However, the current clinical data have yet to uncover the budding effects of SARS-CoV-2 within the CNS and its potentially severe consequences in the coming years. Many studies have confirmed the existence of neurological issues as a long-term effect associated with post-COVID-19 infection. The immediate neurological complications associated with SARS-CoV-2 include encephalitis, encephalopathy, and ADEM [24]. Moreover, one should not ignore the consideration of the prospective negative impacts as the coronavirus could achieve a latent growth phase and later recur to prompt different neurological diseases, such as MS. This review draws insight into the possible mechanisms associated with MS development in SARS-CoV-2 infected people. Since MS is known to occur at any age, the onset usually occurs between 15 and 55 years and COVID-19 is a relatively new disease affecting more adults than young, further studies and investigations are necessary for a better understanding of the possibility of SARS-CoV-2 infection leading to MS. Notably, cytokines and chemokines are modulated in SARS-CoV-2 infection and can interfere in the interplay of the glial cells in MS development. There is a necessity to consider hypoxia-mediated mitochondrial dysfunction and alteration in the phagocytic capacity of microglia/ macrophages in the development of MS. Unwinding MS pathophysiology to the lurking coronavirus could potentially help in the early detection of MS in SARS-CoV-2 infected individuals and result in better medical care.

### Future Perspectives

Many studies have confirmed the existence of neurological issues as a long-term effect associated with post COVID-19 infection. As there is a potential risk of MS pathogenesis as a secondary effect of SARS-CoV-2 disease, future MS development cannot be ruled out; hence, a constant and continuous follow-up of exposed patients would be hugely beneficial. Moreover, this could help better understand and identify factors that may contribute to disease development during the early stages of MS and its staged progression. Consequently, this would provide further insight into effective treatment strategies and intervention and reduce the risk of developing MS or its progression. Furthermore, studies could focus more on the hypoxia and phagocytotic role of microglia during a SARS-CoV-2 infection. Unwinding MS pathophysiology to the lurking coronavirus could potentially help early detect MS in SARS-CoV-2 infected individuals and could result in better medical care.

## Figures and Tables

**Table 1:** Classification, Properties, and CNS Entry Routes of Viruses associated with MS.

Genome	Virus Family	Virus Type	Specifics	Targets	Association with MS	CNS Entry	[REF]
dsDNA	Herpes viridae	Varicella-zoster virus (VZV)	Alpha herpesvirus	neuronal	VZV is frequently detected during the active disease phases of MS	ORN	(Sotelo and Corona, 2011, Marrodan et al., 2019, Tarlinton et al., 2020)
		Herpes simplex virus (HSV-1 and 2)			Viral encephalitis and demyelinating encephalitis	ORN	(Boukhvalova et al., 2020, Marrodan et al., 2019, Tarlinton et al., 2020)
		Cytomegalovirus (CMV)	Beta herpesvirus	non-neuronal (macrophages and B cells)	-CMV seropositivity and MS diagnosis expansion -T-cell driven responses, pneumonia	BBB and BMVEC	(Langer-Gould et al., 2017, Marrodan et al., 2019, Tarlinton et al., 2020)
		Human herpesvirus 6 (HHV-6)		non-neuronal (macrophages and B cells)	-HHV-6 antibody and DNA positivity and MS -HHV-6 proteins have cross reactivity with myelin basic protein, which could contribute to CD8^+^ T cell-mediated oligodendrocyte death	unknown	(Leibovitch and Jacobson, 2014, Marrodan et al., 2019, Tarlinton et al., 2020)
		Epstein–Barr virus (EBV)	Gamma herpesvirus	non-neuronal (macrophages and B cells)	-Infectious mononucleosis, which is caused by delayed primary EBV infection, predisposes MS. -EBV may also contribute to MS pathogenesis indirectly by activating silent human endogenous retrovirus-W.	BBB and BMVEC	(Guan et al., 2019, Langer-Gould et al., 2017, Marrodan et al., 2019, Tarlinton et al., 2020)
dsDNA	Polyomaviridae	Human polyomavirus 2 or John Cunningham virus (JCV)		neuronal	-Progressive multifocal leukoencephalopathy -Risk assessment and monitoring of patients based on JCV seropositivity and antibody titer is necessary in treatment decision for MS	BBB and BMVEC	(Paz et al., 2018, Marrodan et al., 2019, Tarlinton et al., 2020)
ssRNA	Retroviridae	Human endogenous retroviruses (HERVs-H and W)	Gammaretrovirus	non-neuronal (immune cells)	Inflammation, aberrant immune reaction and dysregulated gene expression cellular immune responses	BBB	(Christensen, 2017, Marrodan et al., 2019, Tarlinton et al., 2020)

dsRNA, double-stranded RNA; ssRNA, single-stranded RNA; BBB, blood-brain barrier; BMEV, brain microvascular endothelial cells (BMVEC); ORN: Olfactory Receptor Neuron.

**Table 2:** List of cytokines/chemokines associated with the cytokine storm in SARS-CoV-2 infection association with MS.

Name	MS Associated Function	Reference
IL-2	Plays a role in the loss of immune tolerance. Helps in the proliferation of autoreactive T cells.	(Göbel et al., 2018, Osherov and Milo, 2017)
IL-6	T cell expansion, pro-inflammatory	(Göbel et al., 2018, Ireland et al., 2015, Fiedler et al., 2017)
IL-17	Reduced lesion activity, demyelination in MS	(Göbel et al., 2018, Ghaffari et al., 2017)
IL-10	Anti-inflammatory, Decreases antigen presentation of monocytes and macrophages; Neuroprotective, Decreases prior to relapse and increased during remission	(Göbel et al., 2018, Wei et al., 2019)
IL-7	Lymphocyte development, Increased risk of MS	(Wu et al., 2016, Ghaffari et al., 2017)
IL-8/CXCL8	Chemo-attractant for neutrophils and monocytes, In MS, monocyte recruitment to the CNS	(Lund et al., 2004)
IL-1	Pro-inflammatory, pathogenic role in MS	(Fiedler et al., 2017, Lin and Edelson, 2017, Ghaffari et al., 2017)
GM-CSF	Regulation of microglial functions, stimulation of microglial priming for antigen presentation, pathogenic action in MS	(Aram et al., 2019)
IFN-gamma	Drives inflammation	(Arellano et al., 2015)
TNF-α	Pro-inflammatory	(Fiedler et al., 2017)
TGF-β	Lymphocyte proliferation, differentiation, and survival, protective effect in MS	(Mirshafiey and Mohsenzadegan, 2009)
IP-10/CXCL10	Pathogenesis in MS	(Franciotta et al., 2001)
NO	Dual role- immunomodulatory, Disrupts BBB, demyelination, axonal degeneration	(Smith and Lassmann, 2002)
MCP-1	Pathogenesis in MS	(Franciotta et al., 2001)

## Abbreviations

ACE2	Angiotensin-converting enzyme 2
ADEM	Acute disseminated encephalomyelitis
ARDS	Acute respiratory distress syndrome
BBB	Blood-brain barrier
BDNF	Brain-derived neurotrophic factor
BMEC	Brain microvascular endothelium cells
CNS	Central nervous system
CSF	Cerebrospinal fluid
COVID-19	Coronavirus disease 2019
CCL-CXCL	Chemokines
CLR	C-type lectin receptors
CD8^+^	T cells Cytotoxic T cells
CD4^+^	T cells T-helper cells
FLAIR	Fluid-attenuated inversion recovery
LIF	Leukaemia inhibitory factor
MRI	Magnetic resonance imaging
MHC	Major histocompatibility complex
MMPs	Matrix metalloproteinase
MHV	Mouse Hepatitis Virus
MS	Multiple Sclerosis
NGF	Nerve growth factor
NG2	Nerve/glial antigen 2
PAMP	Pathogen-associated molecular patterns
PRR	Pattern recognition receptors
ROS	Reactive oxygen species
RNA	Ribonucleic acid
SARS-CoV-2	Severe acute respiratory syndrome coronavirus
Th	Helper T-cells
TJ	Tight junctions
TLR	Toll-like receptors
Treg	Regulatory T cells
TNF	Tumour necrosis factor
VEGF	Vascular endothelial growth factor
WHO	World Health Organization
